# ViT-DCNN: Vision Transformer with Deformable CNN Model for Lung and Colon Cancer Detection

**DOI:** 10.3390/cancers17183005

**Published:** 2025-09-15

**Authors:** Aditya Pal, Hari Mohan Rai, Joon Yoo, Sang-Ryong Lee, Yooheon Park

**Affiliations:** 1 Department of Biological Environmental Science, College of Life Science and Biotechnology, Dongguk University, Seoul 04620, Republic of Korea; adityapal88665@gmail.com; 2 School of Computing, Gachon University, Seongnam-si 13120, Republic of Korea; drhmrai@gachon.ac.kr (H.M.R.); joon.yoo@gachon.ac.kr (J.Y.); 3 Department of Food Science and Biotechnology, Dongguk University, Seoul 04620, Republic of Korea

**Keywords:** lung and colon cancer detection, deep learning, ViT-DCNN, medical image classification, self-attention mechanism, performance evaluation

## Abstract

**Background/Objectives:** Lung and colon cancers remain among the most prevalent and fatal diseases worldwide, and their early detection is a serious challenge. The data used in this study was obtained from the Lung and Colon Cancer Histopathological Images Dataset, which comprises five different classes of image data, namely colon adenocarcinoma, colon normal, lung adenocarcinoma, lung normal, and lung squamous cell carcinoma, split into training (80%), validation (10%), and test (10%) subsets. In this study, we propose the ViT-DCNN (Vision Transformer with Deformable CNN) model, with the aim of improving cancer detection and classification using medical images. **Methods:** The combination of the ViT’s self-attention capabilities with deformable convolutions allows for improved feature extraction, while also enabling the model to learn both holistic contextual information as well as fine-grained localized spatial details. **Results:** On the test set, the model performed remarkably well, with an accuracy of 94.24%, an F1 score of 94.23%, recall of 94.24%, and precision of 94.37%, confirming its robustness in detecting cancerous tissues. Furthermore, our proposed ViT-DCNN model outperforms several state-of-the-art models, including ResNet-152, EfficientNet-B7, SwinTransformer, DenseNet-201, ConvNext, TransUNet, CNN-LSTM, MobileNetV3, and NASNet-A, across all major performance metrics. **Conclusions:** By using deep learning and advanced image analysis, this model enhances the efficiency of cancer detection, thus representing a valuable tool for radiologists and clinicians. This study demonstrates that the proposed ViT-DCNN model can reduce diagnostic inaccuracies and improve detection efficiency. Future work will focus on dataset enrichment and enhancing the model’s interpretability to evaluate its clinical applicability. This paper demonstrates the promise of artificial-intelligence-driven diagnostic models in transforming lung and colon cancer detection and improving patient diagnosis.

## Simple Summary

Lung and colon cancer remain two major global health concerns, where early and efficient detection can greatly improve the chances of survival. To address this challenge, we have developed an integrated deep learning model that helps to identify cancerous tissues on medical images. Unlike traditional methods, this model combines two powerful approaches to recognize both overall patterns and very fine details in tissue samples. In this way, it can assist doctors in detecting lung and colon cancer more reliably and with fewer errors. While this approach is still being tested, it shows strong potential to support clinical decision making, reduce delays in diagnosis, and ultimately contribute to better patient care.

## 1. Introduction

Lung and colon cancer are among the most fatal diseases globally, and every year, they take about 1.8 million lives. Lung cancer develops when there is growth of cancerous cells in the lungs, where cells start dividing abnormally to form tumors [1]. This affects essential physiological processes in the lungs, like oxygenation and carbon dioxide elimination. Cancer is often asymptomatic during its early stages, with symptoms only becoming evident during advanced stages, which is why the early detection of lung and colon cancer is critical in increasing life expectancy. Most lung cancer cases are categorized as NSCLC (85%), while SCLC accounts for 15% of cases [2]. Identifying lung cancer as early as possible is imperative, as it may not show symptoms until it has spread to other parts of the body like the bones, liver, or brain. CT scans are one of the most effective medical procedures for the early detection of lung and colon cancer [3], helping clinicians in looking for nodules or masses that might be malignant at a finer volumetric scale. However, performing these scans manually is a tedious task, meaning that clinical changes are sometimes missed in the process. In this regard, DL has transformed medical imaging by allowing the rapid processing and interpretation of big data [4]. With CT scans, it is possible for a physician to overlook signs of a disease at its early stages, while DL algorithms can identify these signs. In the context of lung and colon cancer diagnosis, it has been shown that DL applications can classify a tumor as either benign or malignant, thereby potentially triaging the work of radiologists while enhancing the accuracy of tests [5].

A range of studies have been examined the different approaches to detecting, classifying, and predicting the risk of lung and colon cancer, bringing to the fore the progress made as well as the current challenges involving this topic. A related study [6] previously used an SVM classifier-based method that utilizes MATLAB version 9.8.0.1417392 (R2020a) to detect and predict lung cancer across multiple classes, and the authors obtained detection and forecasting accuracies of 97% and 87%, respectively. However, as the binary treatment employed in this research simplified the complicated predictions, it led to low generalizability. In another study [7], the authors focused on the classification and optimization of lung nodules in CT scans using Linear Discriminant Analysis (LDA) and Deep Neural Networks (DNNs) combined with a Modified Gravitational Search Algorithm, achieving 96.2% sensitivity and 94.56% accuracy, though the model’s generalization to larger and more diverse datasets remained limited. In addition, [8] applied DenseNet-121 with transfer learning on the Chest X-ray 14 and JSRT databases to predict lung cancer, and obtained high accuracy as a result of the deep 121-layer CNN, but the enormous computational complexity of the network became a significant obstacle.

For pulmonary CT images, [9] used a CNN-based feature extraction model with ResNet18 fine-tuning and trained with the Cox model, which resulted in improved risk prediction based on the multimodal features. However, this approach requires large annotated datasets for proper training. Another recent work [10] introduced a deep screen model that integrates multiple deep learning algorithms and successfully improved lung cancer detection in low-dose CT images, yet the implementation of such a large-scale screening system might be quite computationally expensive. To predict contributing factors for delayed cancer diagnosis, [11] used Extreme Gradient Boosting (XGBoost), neural networks, logistic regression and random forests, and found that the most important risk factors are smoking and obesity, but the results were for breast cancer datasets and are not directly applicable to other cancer types.

For colon cancer, [12] used a soft-voting classifier that combines CatBoost, LightGBM, and Gradient Boosting, which resulted in an average accuracy of 0.6583 ± 0.054, while pointing out the necessity of further optimization to achieve better predictive performance. The DNA methylation-based strategy to distinguish primary versus metastatic lung cancer was successfully developed in another study [13] but required sophisticated data processing and high computational power. Moreover, [14] combined the integrated omics signatures with the minimum absolute shrinkage and selection operator methods for the trend classification of lung cancer, which can improve the classification accuracy but suffers from scalability issues in various populations.

Pulmonary cancer detection using deep residual learning with UNet and ResNet for feature extraction was explored by [15], and they achieved 84% efficiency with classifier ensembles. However, the method was shown to have limitations in diagnosing the specific nodule types. In a review of computer-assisted detection (CAD) systems for lung cancer, [16] noted the shortcomings of current classification algorithms for CT, namely their failure to identify all the nodule classes. Finally, [17] suggested an augmented fuzzy cluster-based fuzzy approach combined with morphological thinning for lung cancer prediction via continuous monitoring and enhanced segmentation performance, but it is challenging to incorporate in real-time systems. Overall, these studies demonstrate the remarkable advancements in lung cancer and colorectal cancer detection and prediction based on machine learning and deep learning techniques and, meanwhile, reveal the remaining challenges, including the computational complexity, dataset limitations, model generalization, and real-time application.

This study proposes an advanced ViT-DCNN to enhance lung and colon cancer detection from histopathological images. By leveraging deep learning techniques, this approach aims to improve feature extraction, classification accuracy, and generalization for better early and efficient cancer diagnosis.

The key contributions of this study are as follows:

1. Integrated ViT-DCNN Model: The study proposes a novel integrated model combining Vision Transformer (ViT) with Deformable Convolutional Neural Network (DCNN), leveraging ViT’s self-attention for global contextual feature extraction and DCNN’s adaptive receptive fields for capturing fine-grained, localized spatial details in histopathological images.
2. Superior Performance Metrics: The ViT-DCNN model achieves a test accuracy of 94.24%, precision of 94.37%, recall of 94.24%, and F1-score of 94.23%, outperforming state-of-the-art models like ResNet-152 (92.10%), SwinTransformer (93.80%), and TransUNet (93.90%) across all major metrics.
3. Hierarchical Feature Fusion (HFF): The model introduces an HFF module with a Squeeze-and-Excitation (SE) block to effectively combine global features from ViT and local features from DCNN, enhancing feature representation and improving classification accuracy for lung and colon cancer detection.
4. Robust Data Preprocessing: The study employs comprehensive preprocessing methods, including resizing images to 224 × 224 pixels, min-max normalization, and data augmentation (rotation, zooming, and flipping), to improve model generalization and reduce overfitting on the Lung and Colon Cancer Histopathological Images dataset.
5. Effective Training Strategy: Utilizing the AdamW optimizer with a learning rate of 1 × 10^−5^ and early stopping after five epochs of no validation accuracy improvement, the model ensures efficient training over 50 epochs, achieving stable convergence and high generalizability (validation accuracy of 92.04%).
6. Clinical Relevance: The model’s high precision and recall minimize false positives and negatives, making it a reliable tool for efficient lung and colon cancer detection, with the potential to assist radiologists in clinical settings by improving diagnostic accuracy and patient outcomes.

The remaining sections of the paper are organized as follows. Section 2 outlines the materials and methods used, including data sources, preprocessing techniques, and the proposed framework. Section 3 details the experimental results, providing a comprehensive evaluation of the model’s performance. Section 4 offers a discussion of the findings, addressing their implications and limitations. Finally, Section 5 concludes the study, summarizing the key contributions and suggesting directions for future research.

## 2. Materials and Methods

**Figure 1:**
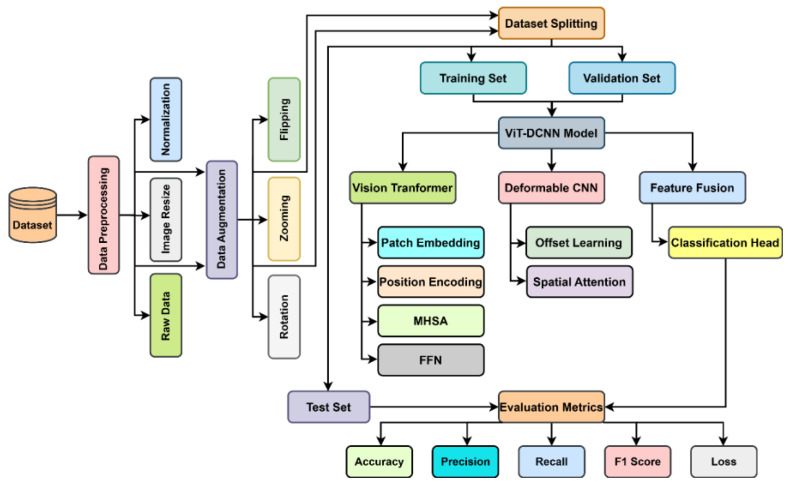
Proposed overall diagram of lung and colon cancer using ViT-DCNN deep learning model.

The present research suggests a hybrid deep learning method that combines a Vision Transformer (ViT) and a Deformable Convolutional Neural Network (CNN) to enhance the detection of lung and colon cancers. The entire procedure encompasses several necessary phases: data collection, preprocessing, model structure design, and performance analysis. The complete workflow applied in this research is shown in Figure 1. In this study, we utilized the Lung and Colon Cancer Histopathological Images Dataset, which comprises histopathological images of normal, benign, and malignant tissue [18]. The dataset was split into a training (80%) and a testing (20%) subset, and the test set was split equally for validation purposes. The images were all resized to 224 × 224 pixels using OpenCV for consistency [19]. Pixel values were normalized between 0 and 1 without losing the RGB color format. Data augmentation techniques, such as rotation and zooming, were used to improve generalization and minimize overfitting. Image labeling was performed automatically according to the respective class directories [20]. For classification, we proposed a hybrid model combining the Vision Transformer (ViT) and the Deformable CNN. The ViT module learns global contextual features by patch embedding, positional encoding, and multi-head self-attention mechanisms [21]. Meanwhile, the Deformable CNN learns local spatial information via adaptive receptive field adjustment. For better representation of features, a Hierarchical Feature Fusion (HFF) mechanism was employed to combine the output of ViT and Deformable CNN using a Squeeze-and-Excitation (SE) block. The end classification was carried out using a softmax layer [22]. The model was trained with the AdamW optimizer, with a learning rate of 1 × 10^−5^. Early stopping was employed to avoid overfitting, halting training when validation accuracy did not show improvement for five consecutive epochs. It was trained for 50 epochs, and its performance was evaluated based on accuracy, precision, recall, and F1 score. It was tested on unseen data after training to evaluate its generalizability. For robustness, the final model was compared with other deep architectures.

### 2.1. Dataset

**Figure 2:**
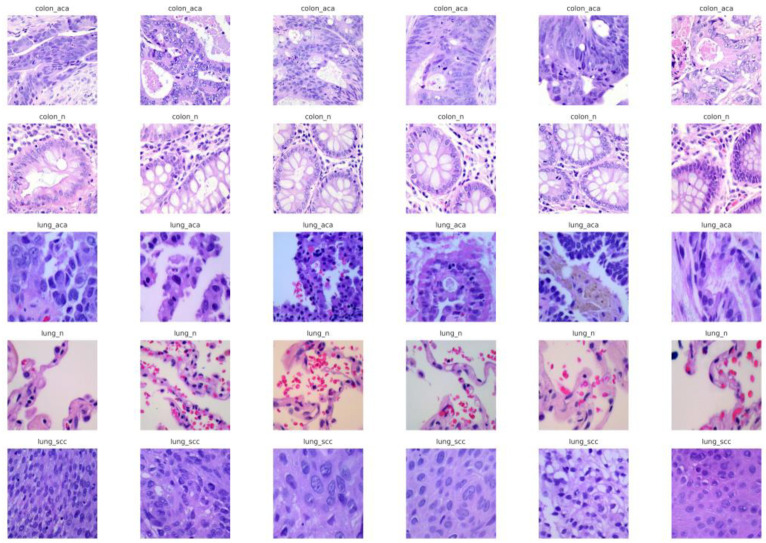
Sample images from the lung and colon cancer histopathology dataset.

The data used in this study was obtained from the Lung and Colon Cancer Histopathological Images Dataset, which provides a large collection of histopathological images suitable for lung and colon cancer diagnosis. The dataset is arranged into five distinct classes, each containing 5000 images: colon adenocarcinoma, colon normal, lung adenocarcinoma, lung normal, and lung squamous cell carcinoma. These categories basically represent the different tissue types, which is beneficial for enabling the development of models capable of accurately distinguishing between normal and malignant tissues [23]. All the images have been resized to a standard input size of 224 × 224 pixels with RGB color channels preserved, ensuring consistency across the dataset and also compatibility with the proposed ViT-DCNN model [24]. Additionally, pixel values have been normalized to the range of 0 to 1 to improve the convergence during model training. For effective learning and evaluation, the dataset was divided into training (80%), validation (10%), and test (10%) subsets using stratified splitting to maintain the class distribution [25,26]. Data augmentation techniques, including random rotations and zooming, were applied during the training to introduce variability and reduce overfitting [27]. This preprocessing approach ensures the dataset was well-prepared for training a deep learning-based model that is capable of robust and accurate classification. Figure 2 shows the sample images of the dataset across five different classes, which are colon adenocarcinoma (colon_aca), colon normal (colon_n), lung adenocarcinoma (lung_aca), lung normal (lung_n), and lung squamous cell cancer (lung_scc).

### 2.2. Data Preprocessing

Data preprocessing is a step that can alter the intricate characteristics of the dataset, and making all the necessary preparations before feeding the data into the machine learning algorithm is crucial. In this study, several methods were used to address the Lung and Colon Cancer Histopathological Images Dataset, which include resizing, normalization, and augmentation.

#### 2.2.1. Image Resizing

Since the dataset contains histopathological images of different sizes and a high resolution, the images were first resized to a size of 224 × 224 pixels, using the reshape function. This standardization was necessary to ensure that the input layer of the ViT-Deformable CNN model could accept each image in a consistent format. It was also noteworthy to mention that resizing helped minimize the number of computations required at the same time; the important features of the images were preserved, which was necessary for feature extraction [28].

#### 2.2.2. Normalization

Subsequently, pixel intensities were scaled to improve the training process and decrease the impact of shadows and similar phenomena resulting from image illumination discrepancies [29]. The original pixel values, which ranged from 0 to 255, were rescaled to a range of 0 to 1 using min-max normalization:

$$x^{\prime }=\frac{x-min\left(x\right)}{max\left(x\right)-min\left(x\right)}$$

where *x* is the original pixel value, *min*(*x*) and *max*(*x*) are the minimum and maximum pixel values in the image, respectively, *x*′ is the normalized pixel value. This normalization process ensured that all images were processed on the same scale, facilitating faster and more stable convergence during model training.

#### 2.2.3. Data Augmentation

To expand the model’s ability to generalize and reduce the issue of overfitting, data augmentation from the training set was used. These transformations altered the images to simulate viewing conditions related to the tissue culture, eliminating the need for physical changes [30]. Augmentation techniques included:

- Rotation: Additionally, images were rotated randomly to values up to 20 degrees. The rotation matrix *R*(*θ*) used for this technique is defined as:

$$R\left(\theta \right)=\left[\begin{array}{cc} cos\left(\theta \right) & -sin\left(\theta \right)\\ sin\left(\theta \right) & cos\left(\theta \right) \end{array}\right]$$

where *θ* represents the angle of rotation.

- Zooming: The model was trained to recognize cancerous patterns at different scales by applying random zoom in/out with a magnification factor up to 20%. This is represented as:

$$Z\left(\alpha \right)=\left[\begin{array}{cc} \alpha & 0\\ 0 & \alpha \end{array}\right]$$

where *α* is the zoom factor applied to the image.

- Flipping: Flipping of images both horizontally and vertically was performed, but the order of the flipping was random, emulating different positions of the tissue samples. These augmentations enlarge the training dataset for the CNN with more diverse images, and in this way, they help the CNN to learn more features.
- Stratified Splitting: To ensure that both the training set and test set had a proper distribution of classes, the data was split into a training set (80%), validation set (10%), and test set (10%) employing the stratified random sampling method. This made sure that each class was fairly divided during each split, taking a number of factors into consideration, particularly where class imbalances exist in routine delivery of medical diagnoses [31]. As a result of data resizing, normalization, augmentations, and stratified splitting, the dataset is well preprocessed for feeding into the deep learning model while improving its performance and also its ability to generalize [32].

### 2.3. Model Design and Description

**Figure 3:**
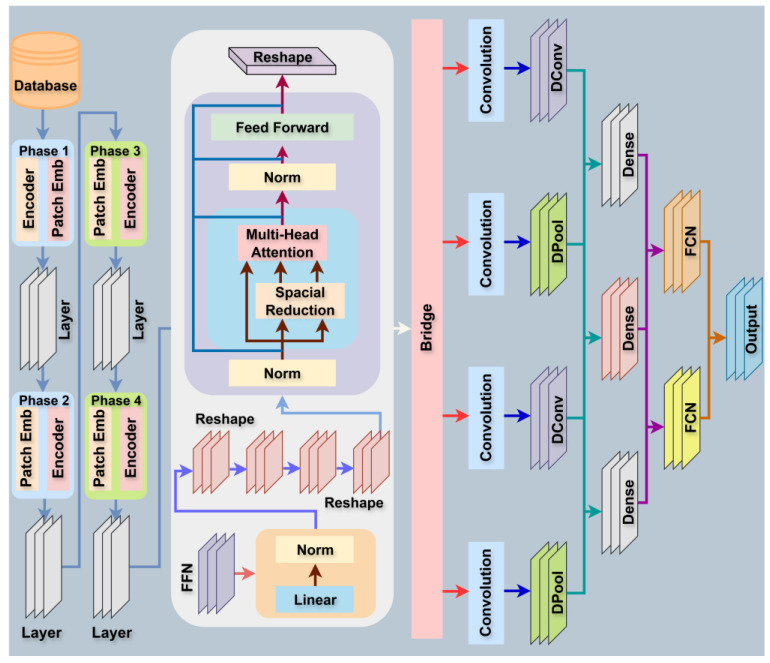
Detailed architecture of ViT-DCNN (Vision Transformer with Deformable CNN).

This work presents a new ViT-DCNN, a hybrid deep learning framework for lung and colon cancer classification from histopathology images [33]. By integrating the global context modeling capacity of Vision Transformers (ViT) and the spatial flexibility of Deformable Convolutions (Deformable CNN), the framework is able to model intricate image structures, especially beneficial in medical imaging where tumors are non-rigid and irregular in shape [34]. Figure 3 represents the detailed architecture of the proposed ViT-DCNN deep learning model. Algorithm 1 presents the proposed ViT-DCN algorithm for lung and colon cancer classification. All notations and their definitions used in the algorithm are listed in the nomenclature section.

#### 2.3.1. Vision Transformer (ViT) Backbone

The Vision Transformer (ViT) backbone is designed to model long-range dependencies by using self-attention mechanisms. The input image is divided into patches, which are then processed through a series of Transformer blocks [35].

Patch Embedding Layer: The input image *X* of size *H* × *W* × *C* (height, width, and channels) is first split into *N* patches of size *P_h_* × *P_w_*. Each patch is flattened into a vector of length *d* (the embedding dimension) and linearly projected into a sequence of embeddings:

$$P_{i}=Flatten\left(X_{i}\right)\cdot W_{emb}+b_{emb},\;\forall i=1,\;\ldots ,N$$

where:

- ${Ρ}_{i}\in R^{d}$ is the *i^th^* patch embedding.
- $W_{emb}\in R^{d\times \left(P_{h}P_{w}C\right)}$ is the learnable projection matrix.
- $b_{emb}\in R^{d}$ is the bias term.
- *X_i_* denotes the *i^th^* image patch.

#### 2.3.2. Positional Encoding

The positional encoding PE*_i_* is added to the patch embeddings to retain spatial information:

$$Z_{i}={Ρ}_{i}+{ΡΕ}_{i},\forall i=1,\ldots ,N$$

where:

- ${ΡΕ}_{i}\in R^{d}$ is the positional encoding, computed as:

$$PE_{i,2k}=sin\left(\frac{i}{1000^{2k/d}}\right),PE_{i,2k+1}=cos\left(\frac{i}{1000^{2k/d}}\right)$$

#### 2.3.3. Multi-Head Self-Attention (MHSA):

The core of the ViT relies on multi-head self-attention (MHSA) to compute dependencies between the patches. Given the sequence of patch embeddings Z = [Z_1_, Z_2_,…, Z_N_], we compute attention as:

$$Attention\left(Q,K,V\right)=softmax\left(\frac{QK^{T}}{\sqrt{d_{k}}}\right)V$$

where:

- $Q\in R^{N\times d_{k}},K\in R^{N\times d_{k}},$ and $V\in R^{N\times d_{v}}$ are the query, key, and value matrices derived from the input embeddings Z.
- *d_k_* is the dimensionality of the keys and queries.
- *d_v_* is the dimensionality of the values.

The multi-head attention mechanism allows the model to learn intricate dependencies by computing attention in multiple subspaces of the input sequence.

#### 2.3.4. Feed-Forward Network (FFN)

After the attention mechanism, the output is passed through a feed-forward network (FFN) to introduce non-linearity [36].

$$FFN\left(Z\right)=\max\left(0,ZW_{1}+b_{1}\right)W_{2}+b_{2}$$

where:

- $W_{1}\in R^{d\times d_{ff}},W_{2}\in R^{d\times d_{ff}}$ are the weights.
- $b_{1},b_{2}\in R^{d_{ff}}$ are the biases.
- *d_ff_* is the size of the hidden layer.

#### 2.3.5. Deformable Convolutional Neural Network (Deformable CNN)

Deformable convolutions are introduced to allow adaptive receptive fields to concentrate on non-regular areas in the image, i.e., the border of tumors. Deformable convolutions introduce a flexible method of learning spatial offsets that allow the network to adjust to the image structure below [37].

#### 2.3.6. Deformable Convolution Layer (DConv)

The standard convolution is modified by introducing offsets for each pixel position. The output *y_ij_* of a deformable convolution is computed as:

$$y_{ij}=\sum_{m=1}^{M}\sum_{n=1}^{N}x_{i+m+∆m_{ij},j+n+∆n_{ij}\cdot w_{mn}}$$

where:

- *x* is the input feature map.
- *w* is the convolution filter.
- Δ*m_ij_*, Δ*n_ij_* are the learned spatial offsets at each location (*i*, *j*).

#### 2.3.7. Offset Learning via Deformable Convolutions

The offsets Δ*m_ij_* and Δ*m_ij_* are learned using a separate convolutional network. The offset generation for a position (*i*, *j*) is computed as:

$$\left[∆m_{ij},∆n_{ij}\right]=Conv\left(F_{ij}\right),F_{ij}\in R^{C}$$

where *F_ij_* is the feature map at location (*i*, *j*), and convolution is applied to generate the offsets through which the model can shift the receptive field to informative regions.

#### 2.3.8. Spatial Attention for Deformable Convolutions

After deformable convolutions, we use a spatial attention mechanism to refine the focus of the model on important features [38]. The spatial attention map *A_ij_* is computed as:

$$A_{ij}=\sigma \left(Conv\left(F_{ij}\right)\right)$$

where:

- σ is the sigmoid activation function.
- Conv(F_ij_) applies a convolutional filter to the feature map F_ij_ to generate attention weights.

The refined feature map *F_refined_* is:

$$F_{refined}=F_{ij}\cdot A_{ij}$$

where · denotes element-wise multiplication.

#### 2.3.9. Hierarchical Feature Fusion (HFF) Module

The Hierarchical Feature Fusion (HFF) module combines the outputs from the ViT and Deformable CNN to take advantage of both global and local feature representations. The fusion is performed by concatenating the feature maps and passing them through a sequence of operations [39].

#### 2.3.10. Feature Concatenation

The feature maps F_ViT_ and F_DConv_ from the ViT and Deformable CNN are concatenated along the channel dimension:

$$F_{concat}=concat\left(F_{ViT},F_{DConv}\right)$$

#### 2.3.11. Squeeze-and-Excitation (SE) Block

A Squeeze-and-Excitation (SE) block is applied to learn channel-wise attention. We first apply global average pooling (GAP) to the concatenated feature map:

$$Ζ=GAP\left(F_{concat}\right)=\frac{1}{HW}\sum_{i=1}^{H}\sum_{j=1}^{W}F_{concat}\left(i,j\right)$$

We then feed the concatenated feature z into a multi-layer perceptron (MLP) to produce channel-wise attention weights:

s=σ(MLP(z))

Finally, the SE block refines the concatenated feature map by applying the attention weights:

$$F_{se}=F_{concat}\cdot s$$

#### 2.3.12. Classification Head

The advanced feature map F_se_ is passed through a global average pooling (GAP) layer to reduce its spatial dimensions, followed by a fully connected (FC) layer with softmax activation to generate class probabilities:

$$P_{c}=Softmax(W\cdot GAP\left(F_{se}\right)+b)$$

where $W\in R^{d\times K}$ is the learned weight matrix, $b\in R^{K}$ is the bias term, and *K* is the number of classes [40].

The softmax function computes the probability of each class *c* as:

$$Softmax\left(z_{c}\right)=\frac{e^{Z_{c}}}{\sum_{k}e^{Z_{k}}}$$

#### 2.3.13. Output Layer

The last hidden layer has a size of 5 neurons because there are 5 classes in the dataset. A softmax activation function is applied to produce probabilities for each class:

$$P\left(y=j|x\right)=\frac{e^{Z_{j}}}{\sum_{k}^{K}e^{Z_{k}}}$$

where *Z_j_* is the input to the neuron corresponding to class *j*, and *K* is the total number of classes (5). This allows the model to predict the probability distribution over the 5 cancer subtypes.

#### 2.3.14. Loss Function

The model is trained using categorical cross-entropy as the loss function since the training is a multi-class classification [41]. The categorical cross-entropy is defined as:

$$L_{cross}=-\sum_{i=1}^{N}\sum_{j=1}^{K}y_{ij}log\left(y_{ij}^{\prime }\right)$$

where:

- *y_ij_* is the binary indicator (0 or 1) if the class label *j* is the correct classification for the observation *i*.
- $y_{ij}^{\prime }$ is the predicted probability of observation *i* being classified as class *j*.

#### 2.3.15. Training Strategy

The model was therefore trained using the Adamax optimizer, with a learning rate of 10^−5^. Early Stopping was used to track validation accuracy and penalize further training after the 5th epoch, when validation accuracy ceased increasing. The model was trained until it reached a maximum of fifty epochs for the training dataset and used a batch of size 16, whereas for the validation and testing dataset, it used a batch of size 8.
**Algorithm 1:** ViT-DCNN (Vision Transformer with Deformable Convolution) for Lung and Colon Cancer Classification1: **Input**: D = {(X_i_, Y_i_)}, α, T, B, θ_ViT_, θ_DConv_, N2: **Initialize**: θ_ViT_, θ_DConv_3: for epoch = 1 to T do4:   for batch = 1 to
$\left(\frac{N}{B}\right)$ do5:         **Extract mini-batch:**6:                 
$\left(X_{batch,}Y_{batch}\right)\leftarrow {\left\{X_{i,}Y_{i}\right\}}_{i=batch}$7:                      **Apply Data Augmentation**:8:                      
$\left(X_{aug,}Y_{aug}\right)\leftarrow Augment\left(X_{batch,}Y_{batch}\right)$9:            **Vision Transformer (ViT) Forward Pass**:10:            **Patch Embedding**:11:                    
$P_{i}=Flatten\left(X_{i}\right)\cdot W_{emb}+b_{emb}$12:            **Positional Encoding**:13:                    Z_i_ = P_i_ + PE_i_14:            **Multi-Head Self-Attention**:15:                   
$Attention\left(Q,K,V\right)=softmax\left(\frac{QK^{T}}{\sqrt{d_{k}}}\right)V$16:         **Feed-Forward Network**:17:                 FFN(Z) = max(0, ZW_1_ + b_1_) W_2_ + b_2_18:          **Deformable Convolution Forward Pass**:19:           **Deformable Convolution**:20:                    
$y_{ij}=\sum_{m=1}^{M}\sum_{n=1}^{N}x_{i+m+∆m_{ij},j+n+∆n_{ij}\cdot w_{mn}}$21:              **Offset Learning**:22:                     
$\left[∆m_{ij},∆n_{ij}\right]=Conv\left(F_{ij}\right),F_{ij}\in R^{C}$23:              **Spatial Attention**:24:                     
$F_{refined}=F_{ij}\cdot A_{ij}$25:          **Hierarchical Feature Fusion (HFF)**:26:              Concatenate Vision Transformer and Deformable CNN Features:27:                     F_concat_ = concat(F_ViT_, F_DConv_)28:              **Squeeze-and-Excitation (SE) Block**:29:                     
s=σ(MLP(z))30:              **Refined Feature Map**:31:                     
$F_{se}=F_{concat}\cdot s$32:          **Prediction and Softmax Activation**:33:              **Global Average Pooling**:34:                     
$Ζ=GAP\left(F_{concat}\right)=\frac{1}{HW}\sum_{i=1}^{H}\sum_{j=1}^{W}F_{concat}\left(i,j\right)$35:              **Softmax Layer**:36:                     
$P_{c}=Softmax(W\cdot z+b)$37:              **Predicted Class**:38:                     
$Y^{\prime }\leftarrow argmax\left(P_{softmax}\right)$39:          **Compute Class**:40:              **Cross-Entropy Loss**:41:                     
$L_{cross}=-\sum_{i=1}^{N}\sum_{j=1}^{K}y_{ij}log\left(y_{ij}^{\prime }\right)$42:              **Gradient Computation**:43:                     
$\nabla \theta_{VIT}L_{cross},\nabla \theta_{DConv}L_{cross}$44:          **Parameter Update (Using AdamW optimizer)**:45:              **Update the Vision Transformer parameters**:46:                     
$\theta_{VIT}\leftarrow \theta_{VIT}-\alpha \cdot \nabla \theta_{VIT}L_{cross}$47:              **Update the Deformable CNN parameters**:48:                    
$\theta_{DComv}\leftarrow \theta_{DConv}-\alpha \cdot \nabla \theta_{DConv}L_{cross}$49:         end for50:  end for51:  **Output**:52:  Trained ViT-Deformable CNN model with updated parameters θ_ViT_ and θ_DConv_

### 2.4. Evaluation Metrics

To evaluate the performance of the proposed ViT-DCNN model for cancer classification, numerous performance measurements have been used. These provide information about the model’s accuracy, stability, and proficiency in determining the type of cancer in different classes.

***Accuracy***: Accuracy is an elementary measure used to compare the percentage of correctly classified instances with the total instances [42]. It is calculated as follows:

$$Accuracy=\frac{TP+TN}{TP+TN+FP+FN}$$

where:

- *TP* (True Positive) is the number of correctly predicted positive instances (lung and colon cancer cases).
- *TN* (True Negative) is the number of correctly predicted negative instances (non-cancer cases).
- *FP* (False Positive) is the number of incorrectly predicted positive instances.
- *FN* (False Negative) is the number of incorrectly predicted negative instances.

A higher accuracy therefore means that the model has a better ability to distinguish lung and colon cancer cases from non-cases.

***Precision***: Precision evaluates the accuracy of positive predictions and indicates the model’s ability to avoid false positives [43]. It is calculated using the formula:

$$Precision=\frac{TP}{TP+FP}$$

A high precision means that the model is not generating many false positive predictions; thus, it is an important measure, especially in medical applications.

***Recall***: Recall, also referred to as sensitivity or the true positive fraction, refers to a model’s capacity to capture all positive cases [44]. It is defined as:

$$Recall=\frac{TP}{TP+FN}$$

This metric is particularly important in medical applications, for example, where a missed positive example can have dire consequences.

***F1 Score***: The F1 score is obtained by the harmonic average of precision and recall when the two are equal. It is particularly helpful when working with disproportionate datasets where one of the classes is larger than the other [45]. The F1 score is computed as:

$$F1Score=2\cdot \frac{Precision\cdot Recall}{Precision+Recall}$$

An F1 score operating at a high level shows a combination of both precision and recall.

***Loss***: Loss measures the discrepancy between the calculated output and the actual labels. It plays a critical role in fine-tuning the model during training and is described in full below [45]. In the context of classification tasks, the most commonly used loss function is the categorical cross-entropy loss, defined as:

$$Loss=-\sum_{i=1}^{N}\sum_{j=1}^{K}y_{ij}log\left(y_{ij}^{\prime }\right)$$

where:

- *y_ij_* is the binary indicator (0 or 1) if the *class* label *j* is the correct classification for the observation *i*.
- $y_{ij}^{\prime }$ is the predicted probability of observation *i* being classified as class *j*.

## 3. Experimental Result

The proposed ViT-DCNN model was thoroughly validated on the dataset containing images of different forms of cancer. The following values attributed to each model were utilized in this comparative assessment: accuracy, precision, recall rate, F1 score, and loss. Overall, these parameters provide a complete estimation of the model, its reliability, and its accuracy for classification and detection of cancer types in a crucial clinical setting.

**Figure 4:**
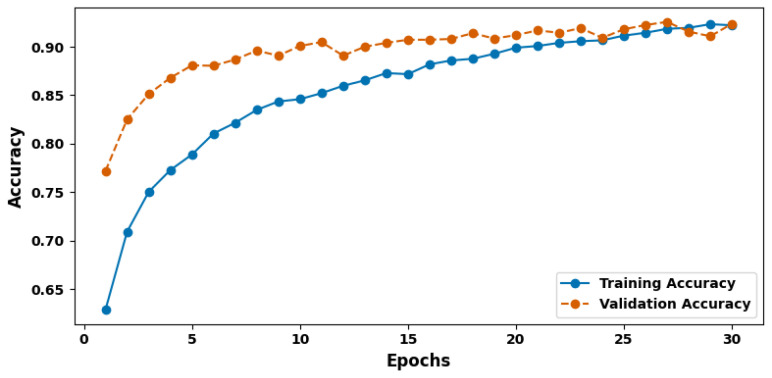
Training and validation accuracy plot.

Figure 4 shows the training and validation accuracy plot. Accuracy is one of the simplest and most significant measures of performance, presenting the overall ratio of correctly classified elements to the total number of elements considered. In this experiment, the model underperformed on the training dataset and had a test data set accuracy of 92.88%. This high degree of specificity means that the model successfully classifies a very large proportion of the instances and confirms that it has learned to identify the varying classes into which the dataset has been split. When scaled up for medical image analysis, where diagnoses can dictate patient outcomes, such a result is particularly encouraging.

**Figure 5:**
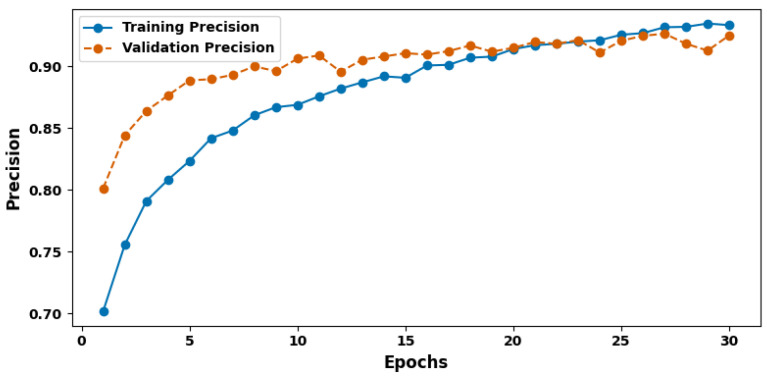
Training and validation precision plot.

Figure 5 shows the training and validation precision plot. The latter is the frequently used precision that defines the share of true positive predictions among all positive predictions made by the model. In this study, the model’s accuracy percentage was 93.96%. A high value of precision means that there is a small incidence of Type II errors when the model predicts the presence of cancer, with the precision being approximately about 94%. There is nothing more important than a precision score in the medical field, where false positives often result in unnecessary fear and additional rounds of operations. It demonstrates feasibility by showing that high-confidence judgments are indicative of a positive diagnosis in practice, thereby increasing the practical utility of the model in clinical application.

**Figure 6:**
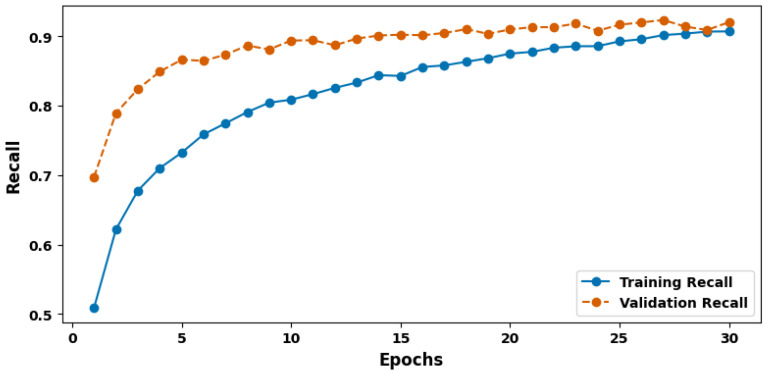
Training and validation recall plot.

Figure 6 shows the training and validation recall plot. The metric of recall is equally critical, as it assesses the model’s ability to identify all actual positive cases within the dataset. The model exhibited a recall of 91.42%, suggesting that it successfully identifies around 91% of all true cancer instances. This high recall rate is closely linked to the model’s ability to minimize false negatives, a crucial aspect of cancer diagnosis. Controlling the recall means that the diagnosis of cancer has to be made as early and accurately as possible to ensure that the treatment outcome is successful and the patient’s condition improves, which is highly desirable for any diagnostic tool used in oncology.

**Figure 7:**
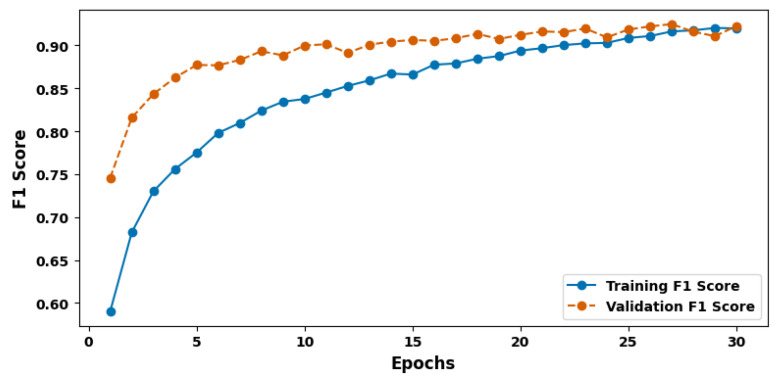
Training and validation F1 score plot.

Figure 7 shows the training and validation F1 score plot. To further evaluate the model’s performance, the F1 score was calculated, which combines the measures of precision and recall into a single value at 92.67%. It reveals that the chosen score has equal measures of precision and recall, thus supporting the idea that the model will be capable of omitting both false positive and false negative cases. The F1 score is especially useful in situations with skewed classes, which are common for medical datasets.

**Figure 8:**
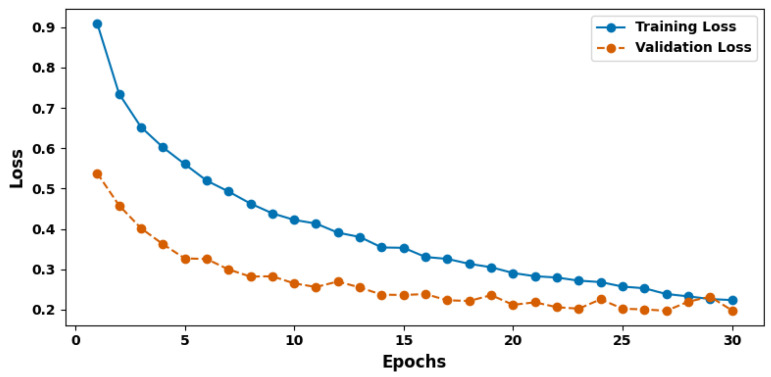
Training and validation loss plot.

Figure 8 shows the training and validation loss plot. The measured loss obtained during training provides insights into how the model’s predictions compare with the actual labels. The values set in the model resulted in a loss of 0.2041, a value that is preferred for learning. The smaller the loss value, the more accurate the model’s predictions are and the closer they are to the true labels, indicating better parameter tuning during training. Validation metrics offer an even further measure of the accuracy and thus the generalization ability of the model. For the validation accuracy, the above built model gave an accuracy of 92.04%, which means that the model can replicate accuracy on unseen data. In the case of validation, the accuracy was noted as 92.06%, and the recall was noted as 91.80%. The F1 score for validation was 91.93%, and the loss validation was 0.2005. These validation results are very similar to the training metrics, suggesting that the learning that takes place is both efficient and capable of generalizing to new instances. Thus, validation is a critical aspect in determining how well the model can be employed in real-world situations, especially for hospitalized patients where differential diagnosis is of high value.

### 3.1. Confusion Matrix for Test Set

**Figure 9:**
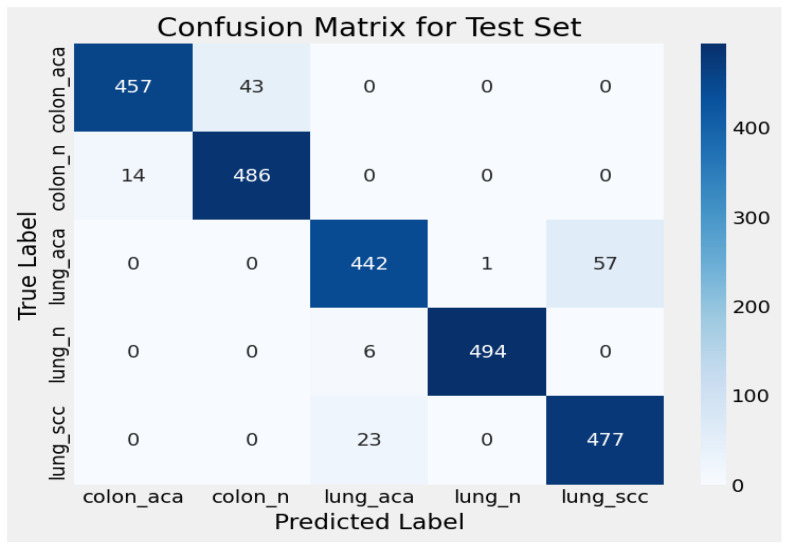
Test set confusion matrix.

The confusion matrix for the test set reveals the performance of the classification model across five different classes, which are colon adenocarcinoma (colon_aca), colon normal (colon_n), lung adenocarcinoma (lung_aca), lung normal (lung_n), and lung squamous cell cancer (lung_scc). The matrix also presents the degree to which the model was able to correctly classify each of the classes and some instances where it performed poorly. In colonic adenocarcinoma (colon_aca), 457 instances were correctly classified, and 43 samples were mistakenly classified as colon normal (colon_n). This shows that the model slightly fails to accurately separate between these two classes, although no other class misclassification was observed for colon_aca. The true number in the colon normal (colon_n) group was correctly identified by the model with 486 cases. Nevertheless, 14 samples were assigned to colonic adenocarcinoma (colon_aca) incorrectly. This suggests that the patterns in these two classes may overlap, which would be a focus for further model improvement. Out of 442 samples of lung adenocarcinoma, a few were misclassified: 57 as lung squamous cell carcinoma (lung_scc) and 1 as lung normal (lung_n). Such misclassification patterns indicate that the algorithm has slightly more difficulties in distinguishing between (lung_aca) and (lung_scc). The results of normal lung (lung_n) show high accuracy, with 494 samples of the lung normal class being correctly classified, but only 6 samples belonging to lung adenocarcinoma (lung_aca) were misclassified. This result means that the model is able to clearly differentiate normal lung from the other classes. Lastly, for lung squamous cell carcinoma (lung_scc), 477 samples were correctly assigned, whereas 23 samples were misclassified as lung adenocarcinoma (lung_aca). As in (lung_aca), the split of these two types of lung cancers is something that may be further refined. In summary, the overall performance of the model is satisfactory. However, the distinction between colonic adenocarcinoma and normal colon, and between lung adenocarcinoma and lung squamous cell carcinoma, needs to be improved. Fine-tuning the model or implementing additional data preprocessing techniques may help reduce these misclassifications. Figure 9 shows the confusion matrix for the test set.

### 3.2. Model Evaluation Metric Comparison

**Figure 10:**
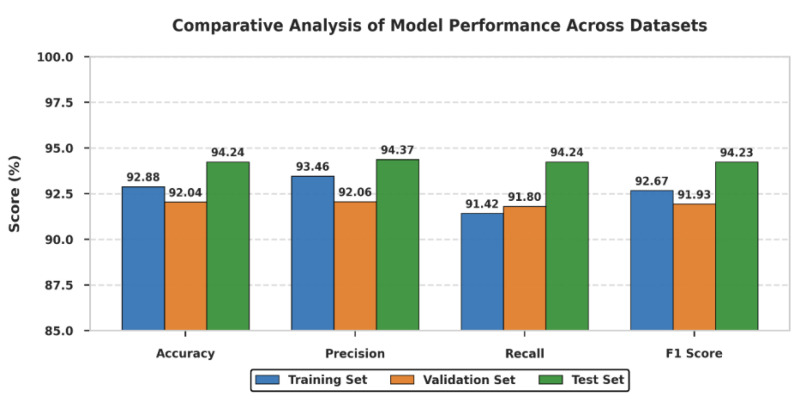
Comparison of evaluation metrics.

The assessment of the precision, recall, F1 score, and accuracy indicates mixed performance in the training, validation, and test groups. Table 1 represents the comparative analysis of key metrics. For the training set, the precision was 93.46%, implying that the percentage of actual positives out of the total number of positive predictions for the training was 93.46%. The recall was found to be 91.42%, which means that the model accurately flagged 91.42% of the actual positives. This study gave a fairly good F1 score of 92.67%, which is the harmonic mean of the precision and the recall values obtained. As a result, while training the model, an accuracy of 92.88% was attained, proving the generally satisfactory performance of the model on the training material. Similarly, the validation set had a slightly lower precision of 92.06% of the positive cases that the model predicted were right. The accuracy was found to be 91.80%, which shows the capacity of the model to correctly identify actual positives. Based on the above accuracy measures and the F1 score of 91.93%, it is clear that the output is well balanced between precision and recall. The model achieved 92.04% accuracy on the validation set as a result of cross-validation, which presents the ability of the model to perform well on new unseen data. In the test set, the model also demonstrated good performance, with a precision of 94.37%, indicating that it was correct most of the time when predicting positive outcomes. For the actual positives, the recall was calculated as 94.24% of actual positives retrieved. The F1 score obtained as a result was 94.24%, which is quite acceptable, proving that the model possesses both adequate precision and recall rates. The accuracy for the test set was 94.24%, demonstrating consistency with unseen data at the same level as the previous test set. All in all, the performance of the model in both the training and test sets is strong and rather stable in terms of precision, recall, F1 score, and accuracy, with the best performance in the test set, where all measurements are around 94%. This implies that the model has been trained well, and it will perform well when applied to new data without compromising on accuracy. Figure 10 represents the evaluation metric comparison.

**Figure 11:**
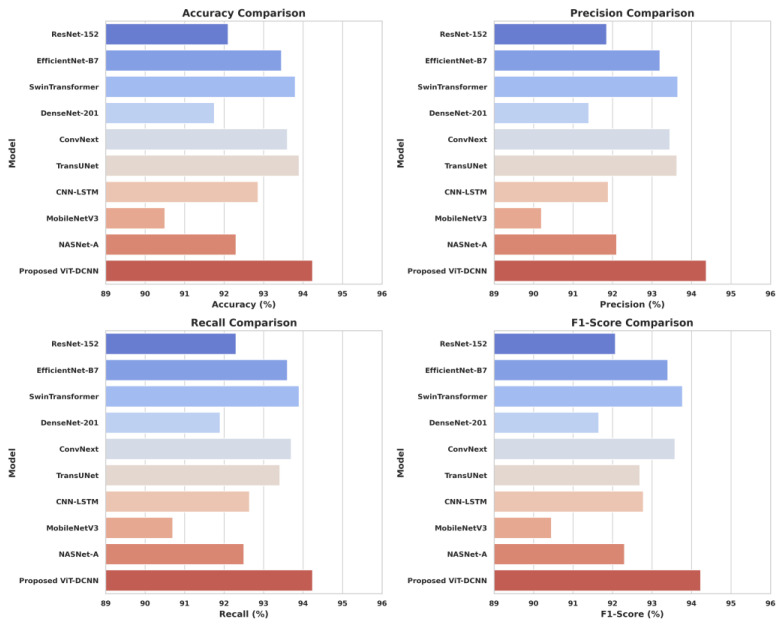
Visualized performance of the proposed ViT-DCNN model compared with other SOTA (state-of-the-art) models.

The performance of our proposed ViT-DCNN model was compared with several well-known deep learning models to evaluate its effectiveness in cancer detection. As shown in Table 2, the ViT-DCNN model achieved the highest results across all major metrics: accuracy, precision, recall, and F1-score. It reached an accuracy of 94.24%, which is higher than all state-of-the-art models. The closest competitor was TransUNet with 93.90% accuracy, followed by SwinTransformer (93.80%) and ConvNext (93.60%). In terms of precision, our model scored 94.37%, meaning it was very good at correctly identifying only the actual cancer cases and avoiding false positives. This was slightly higher than SwinTransformer (93.65%) and EfficientNet-B7 (93.20%). For recall, which indicates how well the model identified all actual cancer cases, the ViT-DCNN achieved 94.24%, again the highest among all models. The F1-score of our model was also the best at 94.23%, showing a strong balance between precision and recall. SOTA models like CNN-LSTM, NASNet-A, and ResNet-152 demonstrated lower F1-scores, indicating that either they failed to detect more actual cases or were making more wrong predictions than our model. To conclude, the above comparison shows that the ViT-DCNN model outperforms traditional CNN-based models (like ResNet and DenseNet), hybrid models (like CNN-LSTM), and even advanced transformer-based models (like SwinTransformer and TransUNet). These results highlight the advantages of combining Vision Transformers with Deformable Convolutions, enabling the model to understand both the global context and fine details in medical images. The strong performance of our proposed model makes it a reliable and powerful tool for helping doctors and medical practitioners in the efficient diagnosis of lung and colon cancer. Figure 11 represents the comparative analysis of the proposed model with the state-of-the-art models.

While the proposed ViT-DCNN model delivers outstanding accuracy in classifying lung and colon cancer and their subtypes, there remain opportunities to refine the model further. Its hybrid architecture requires higher computational resources, and its interpretability could be enhanced to provide clinicians with more transparent decision support. Additionally, the model’s robustness could be further validated across the datasets with varied staining protocols or imaging equipment. These considerations still highlight promising directions for future work, while the following results demonstrate the strong diagnostic performance and the reliability of the proposed model.

## 4. Discussion

With the rise in applications of deep learning and machine learning methods for lung and colon cancer detection, it has been shown that these approaches can achieve significant improvements in predictive performance over conventional methods. Previous studies have used a range of methods, from SVM classifiers, CNNs, ResNet variants, and hybrid pipelines, which have enabled the reasonable delivery of metrics like high accuracy, precision, recall, F1 score, and others. For example, SVM-based methods for analyzing lung cancer image analysis resulted in a precision of 94.68% and recall of 92.84% [6], and Raman spectroscopy together with 1D-CNN classifiers had an accuracy of 94.5% for multi-cancer detection [46]. Similarly, ResNet-based architectures and transfer learning techniques on chest imaging datasets displayed moderate accuracies (74–76%) but highlighted the challenges in terms of computational complexity and demands of large annotated datasets [8,9].

Techniques to identify colon cancer using Serum Raman spectroscopy and a machine learning algorithm have been shown to be accurate at 95% [47], and deep learning methods comparing optimizers, including SGD, Adamax, AdaDelta, RMSprop, Adam, and Nadam with CNN models, were found to be 90% accurate with a precision of 89%, recall of 87%, and an F1 score of 87% [48]. Soft-voting ensemble classifiers showed moderate accuracy (~65–70%) [12], suggesting the need for further optimization to enhance generalizability. Deep learning models (VGG16, VGG19, InceptionV3, and ResNet-50) on the histopathological slides attained high accuracy (~96.5%) [15] but rarely provided adequate interpretability, which is essential for clinical adoption. Hybrid pipelines, combining SqueezeNet-based feature extraction with traditional classifiers, generated high predictive performance accuracy of 92.9% but were limited by relatively small datasets [49]. Similarly, SVM models obtained using TPOT gave good results for early lung cancer classification (accuracy 91.77%), but false-positive rates were still a concern [50].

In this study, the proposed ViT-DCNN model, by combining Vision Transformer with Deformable CNN, showed promising performance for both lung and colon cancer detection (accuracy: 94.24%, precision: 94.37%, recall: 94.24%, F1 score: 94.23%). The proposed architecture effectively combines the global context modeling of Vision Transformers with the spatially localized feature extraction of deformable CNNs, which strikes a balance between accuracy and computational efficiency. Furthermore, the proposed model provides potential for improved interpretability through future integration of explainable AI paradigms, addressing one of the main limitations of past studies. Overall, this research indicates that deep learning models, especially those combining multiple architectural benefits, have great potential to enhance clinical decision making on cancer detection and classification and underscores the need for external validation and explainability for wider clinical use. Table 3 represents the comparative analysis for lung and colon cancer detection based on machine learning and deep learning.

### SWOT Analysis of Proposed ViT-DCNN Model

The SWOT analysis of the proposed ViT-DCNN model provides insight into the strong potential of the model for cancer classification tasks. Among other key strengths, the hybrid architecture of Vision Transformer and Deformable CNN is indeed highly efficient in capturing global context as well as fine-grained local features from histopathology images. This capability is further supported by the strong preprocessing techniques like normalization, augmentation, and stratified data, which is split to improve the input quality and guarantee proper model generalization. Further, Hierarchical Feature Fusion and the Squeeze-and-Excitation blocks reinforce feature representations, and the use of optimized training strategies with the Adamax and early stopping ensures stable convergence and avoids overfitting.

Although the model has been validated on a single dataset, this provides a strong and robust baseline and opens up opportunities for future validation in multi-dataset and multicenter studies. The Transformer backbone is computationally intensive, which suggests that it is ready for scaling up to the large clinical datasets. In addition, model interpretability is an emerging area of research and thus offers opportunities to incorporate explainable AI techniques in the future.

In terms of opportunities, the model could be applied to other histopathology datasets to further improve generalizability and clinical utility. It also shows great promise for integration into actual digital pathology workflows, especially for early detection of cancer and treatment planning. Moreover, the hybrid approach can promote research on explainable architectures to make the system more transparent and acceptable for clinical use, whereas the preprocessing strategy itself could be used as a standardized pipeline for other applications in medical imaging.

Finally, external factors, which are usually expressed in the form of threats, can be viewed as positive incentives. For example, regulatory validation is necessary for clinical translation, which encourages collaborative studies in medical institutions. In addition, the enormous speed of AI development provides a source of constant model improvement. Inter-institutional data heterogeneity across healthcare institutions also provides an opportunity for investigating domain adaptation and federated learning, which would further enhance the robustness and generalizability of the model across different clinical settings.

## 5. Conclusions

Therefore, the approach, along with the evaluation of our proposed ViT-DCNN model for detecting and classifying lung and colon cancer, has marked a step toward the development of AI-enabled healthcare solutions. The findings of the study also demonstrate the specific performance of the models, which have a high level of accuracy, making it possible to identify lung and colon cancer cases with an accuracy of 92.88%, an F1 score of 92.67%, a precision of 93.96%, and a recall of 91.42%, based on the medical imaging data. In the test set too, the model had a good performance, with a precision of 94.37%, indicating that the model got it right most of the time when it predicted positive. For the actual positives, the recall was calculated as 94.24% of actual positives retrieved. The F1 score, which was obtained as a result, was 94.24%, which is quite acceptable, proving that the model possesses both good precision and recall rates. The accuracy for the test set was 94.24%, demonstrating consistency with unseen data at the same level as the previous test set. Additionally, the proposed ViT-DCNN model outperformed several state-of-the-art models, including ResNet-152, EfficientNet-B7, SwinTransformer, DenseNet-201, ConvNext, TransUNet, CNN-LSTM, MobileNetV3, and NASNet-A, across all major evaluation metrics, showcasing its superiority in detecting lung and colon cancer. These results imply the effectiveness of the proposed model to assist radiologists and clinicians for early and accurate diagnosis of lung and colon cancer with consequent prompt therapeutic management. There is thus evidence of good model learning and generalization, and the relatively low loss value of 0.2041 is indicative of the model’s stability. Since lung and colon cancer continue to be a significant health menace leading to cancer-related deaths globally, adoption of such advanced AI tools such as our proposed ViT-DCNN model will enhance diagnostic accuracy, reduce the workload on healthcare systems, and benefit patients. For future work, more data should be collected for different lung and colon cancer subtypes, and the model should be evaluated on multiple imaging datasets to improve generalizability. In addition, applying explainability methods to highlight the basis of model predictions will be essential for building trust among healthcare professionals and it will also lead to clinical interpretability. Beyond these directions, the potential applications of the proposed ViT-DCNN model extend across broader healthcare settings. The framework could be adapted to other histopathological datasets, which enables its use in the detection of a variety of cancers and also related diseases. Integration into computer-aided diagnostic systems within hospitals could provide radiologists and pathologists with efficient, reliable second opinions, reducing diagnostic delays and minimizing errors. The model also holds promise for deployment in low-resource environments where expert pathologists are scarce, thereby expanding access to quality diagnostics on a global scale. Furthermore, coupling this approach with cloud-based platforms and telemedicine services would enhance scalability and will also ensure remote accessibility. Integration with electronic health records (EHRs) and clinical decision support systems could simplify the diagnostic workflows and adjust them to the longitudinal tracking of tissue samples so that the response to the treatment would be monitored over time. In conclusion, this paper shows not only the technical contribution of the proposed ViT-DCNN model to the process of cancer detection improvement but also its potential contribution to clinical practice. Combining the highly predictive capability with the opportunities of the practical applications, this work emphasizes the growing contribution made by AI-based models to efficient cancer diagnoses, improved patient outcomes, and the future of intelligent healthcare delivery.

## Figures and Tables

**Table 1:** Comparative analysis on key metrics used in this research.

Evaluation Criteria	Training Set	Validation Set	Test Set
**Accuracy (%)**	92.88	92.04	94.24
**Precision (%)**	93.46	92.06	94.37
**Recall (%)**	91.42	91.80	94.24
**F1 Score (%)**	92.67	91.93	94.23

**Table 2:** Comparative analysis of the proposed ViT-DCNN model with the state-of-the-art methods.

Model	Accuracy (%)	Precision (%)	Recall (%)	F1-Score (%)
**ResNet-152**	92.10	91.85	92.30	92.07
**EfficientNet-B7**	93.45	93.20	93.60	93.40
**SwinTransformer**	93.80	93.65	93.90	93.77
**DenseNet-201**	91.75	91.40	91.90	91.65
**ConvNext**	93.60	93.45	93.70	93.58
**TransUNet**	93.90	93.63	93.41	92.69
**CNN-LSTM**	92.86	91.89	92.64	92.78
**MobileNetV3**	90.50	90.20	90.70	90.45
**NASNet-A**	92.30	92.10	92.50	92.30
**Proposed ViT-DCNN**	**94.24**	**94.37**	**94.24**	**94.23**

**Table 3:** Comparative analysis of machine learning and deep learning methods for lung and colon cancer detection.

References	Purpose	Method	Key Metrics (%)	Challenges
[6]	Multi-class lung cancer detection and prediction	SVM classifier-based approach using MATLAB version 9.8.0.1417392 (R2020a) for image processing	Precision: 94.68 Recall: 92.84	Binarization may oversimplify complex predictions, limiting generalizability
[46]	Develop low-cost, non-destructive cancer screening using serum Raman spectroscopy	Raman spectra database with 1D-CNN for classifying gastric, colon, rectal, and lung cancers	Accuracy: 94.5 Precision: 94.7 Recall: 94.5 F1 Score: 94.5 Kappa Coefficient: 93	CNN interpretability remains limited
[8]	Lung cancer prediction using chest imaging	DensNet-121 with transfer learning on Chest X-ray 14 and JSRT databases	Accuracy: 74.43 ± 6.01 Specificity: 74.96 ± 9.85 Sensitivity: 74.68 ± 15.33	Computational complexity due to deep network layers
[9]	Risk assessment and estimation using pulmonary cancer CT images	CNN for feature extraction, fine-tuning ResNet18, and training with Cox model	AUC: 76 F1 Score: 63 Matthew Correlation Coefficient: 42	Requires large, annotated datasets to train effectively
[47]	Rapid colon cancer detection with tumor markers and spectroscopy	Serum Raman spectroscopy with ELISA for tumor markers and machine learning for classification	Accuracy: 95	Limited biomarker correlation explored
[48]	Explore deep learning techniques for colon cancer classification	Compared optimizers such as SGD, Adamax, AdaDelta, RMSprop, Adam, and Nadam on CNN models	Accuracy: 90 Precision: 89 Recall: 87 F1 Score: 87	Optimizer performance varied between datasets
[12]	Colon cancer risk prediction	Soft-Voting classifier with CatBoost, LightGBM, and Gradient Boosting	Accuracy: 65.8 ± 5.4 Recall: 69.5 ± 6.8 F1 Score: 67.3 ± 2.5 Precision: 66.2 ± 8.3	Requires optimization for increased accuracy
[15]	Improve recognition accuracy for lung cancer from histopathological slides	Evaluated six deep learning models including CNN, CNN-GD, VGG16, VGG19, InceptionV3, and ResNet-50	Accuracy: 96.52 Precision: 92.14 Sensitivity: 93.71 Specificity: 92.91 F1 Score: 94.21	Algorithms lacked sufficient explainability
[49]	Enhance lung cancer detection using hybrid deep learning and machine learning pipeline	Used SqueezeNet for feature extraction followed by machine learning classifiers on chest CT scans	Accuracy: 92.9 Precision: 92.8 Recall: 92.9 F1 Score: 92.8	Dataset size was relatively small
[50]	Improve early lung cancer classification using TPOT SVM	CT images processed through AMF preprocessing and M-SegNet segmentation followed by feature extraction and TPOT SVM classifier	Accuracy: 91.77 True Positive Rate: 94.79 False Positive Rate: 11.24	False positive rate remained comparatively high
**Proposed ViT-DCNN model**	To revolutionize efficient and accurate detection of lung and colon cancers by leveraging advanced deep learning architectures for improved clinical decision making	Integrated (ViT-DCNN) Vision Transformer with Deformable CNN for feature extraction and classification	Accuracy: 94.24 Precision: 94.37 Recall: 94.24 F1 Score: 94.23	Offers opportunities for broader external validation and enhanced interpretability through future explainable AI integration

## Data Availability

The dataset used in this work is publicly available and may be freely downloaded from the given link: LC25000 Dataset: https://www.kaggle.com/datasets/andrewmvd/lung-and-colon-cancer-histopathological-images (accessed on 11 January 2025).

## Nomenclature

**Symbols**	**Description**
***D***	Dataset consisting of image-label pairs $\left\{\left(X_{i},Y_{i}\right)\right\}$
***α***	Learning rate
***T***	Total number of epochs
***B***	Batch size
***θ_ViT_***	Weights of the Vision Transformer model
***N***	Total number of samples
***θ_DConv_***	Weights of the Deformable Convolutional model
***X_batch_***	A mini-batch of input images extracted for training
***Y_batch_***	Corresponding labels of the images in the mini-batch $X_{batch\;\;\;}$
***X_aug_***	Augmented version of the mini-batch images $X_{batch\;\;\;}$
***Y_aug_***	Labels corresponding to the augmented images $X_{aug\;}$
***P_i_***	Patch embedding of input image $X_{i\;\;\;}$
***W_emb_, b_emb_***	Patch embedding weight and bias
***Z_i_***	Positional encoding of input patches
***PE_i_***	Positional encoding vector
***Q, K, V***	Query, Key, and Value matrices in Multi-Head Self-Attention
***d_k_***	Dimension of the key vectors in attention mechanism
**Attention (** ***Q*** **, ** ***K*** **, ** ***V*** **)**	Multi-Head Self-Attention mechanism
***W*** **_1_, ** ***W*** **_2_, ** ***b*** **_1_, ** ***b*** **_2_**	Weights and biases for Feed-Forward Network (FFN) in ViT
***y_ij_***	Output of the Deformable Convolution at position (i,j)
$x_{i+m+∆m_{ij},j+n+∆n_{ij}\cdot w_{mn}}$	Input feature at position $\left(i+m,j+n\right)$ with learned offsets
***w_mn_***	Deformable convolution kernel weights
**[Δ*m_ij_*, Δ*n_ij_*]**	Learned spatial offsets in deformable convolution
***F_ij_***	Input feature map at position (i,j)
***A_ij_***	Attention weight for spatial feature refinement
***F_refined_***	Output of spatial attention mechanism
***F_ViT_***	Features extracted from Vision Transformer
***F_DConv_***	Features extracted from Deformable CNN
***F_concat_***	Concatenated feature map from ViT and Deformable CNN
***s***	Squeeze-and-Excitation scaling factor
σ(·)	Activation function (Sigmoid in SE block)
**F_se_**	Refined feature map after SE block
***Z***	Global Averaged Pooled (GAP) feature vector
***H*, *W***	Height and width of the feature map
***GAP* (*F*)**	Global Average Pooling operation
***P_c_***	Probability distribution over classes (softmax output)
***W, b***	Weights and bias for classification layer
$\hat{Y}$	Predicted class label (argmax of softmax probabilities)
***L_cross_***	Cross-Entropy loss function
***θ_VIT_L_cross_***	Gradient of the loss with respect to Vision Transformer weights
***θ_DConv_L_cross_***	Gradient of the loss with respect to Deformable CNN weights
